# Adaptation and learning as strategies to maximize reward in neurofeedback tasks

**DOI:** 10.3389/fnhum.2024.1368115

**Published:** 2024-03-25

**Authors:** Rodrigo Osuna-Orozco, Yi Zhao, Hannah Marie Stealey, Hung-Yun Lu, Enrique Contreras-Hernandez, Samantha Rose Santacruz

**Affiliations:** ^1^Department of Biomedical Engineering, University of Texas at Austin, Austin, TX, United States; ^2^Department of Electrical and Computer Engineering, University of Texas at Austin, Austin, TX, United States; ^3^Institute for Neuroscience, University of Texas at Austin, Austin, TX, United States

**Keywords:** brain-computer interface, neural manifold, reinforcement learning, neurofeedback, adaptation, dimensionality reduction

## Abstract

**Introduction:**

Adaptation and learning have been observed to contribute to the acquisition of new motor skills and are used as strategies to cope with changing environments. However, it is hard to determine the relative contribution of each when executing goal directed motor tasks. This study explores the dynamics of neural activity during a center-out reaching task with continuous visual feedback under the influence of rotational perturbations.

**Methods:**

Results for a brain-computer interface (BCI) task performed by two non-human primate (NHP) subjects are compared to simulations from a reinforcement learning agent performing an analogous task. We characterized baseline activity and compared it to the activity after rotational perturbations of different magnitudes were introduced. We employed principal component analysis (PCA) to analyze the spiking activity driving the cursor in the NHP BCI task as well as the activation of the neural network of the reinforcement learning agent.

**Results and discussion:**

Our analyses reveal that both for the NHPs and the reinforcement learning agent, the task-relevant neural manifold is isomorphic with the task. However, for the NHPs the manifold is largely preserved for all rotational perturbations explored and adaptation of neural activity occurs within this manifold as rotations are compensated by reassignment of regions of the neural space in an angular pattern that cancels said rotations. In contrast, retraining the reinforcement learning agent to reach the targets after rotation results in substantial modifications of the underlying neural manifold. Our findings demonstrate that NHPs adapt their existing neural dynamic repertoire in a quantitatively precise manner to account for perturbations of different magnitudes and they do so in a way that obviates the need for extensive learning.

## 1 Introduction

Understanding how new motor skills are acquired and lost is crucial for the development of effective neuroprosthetic devices for mitigating the impacts of aging and neurodegenerative conditions, as well as for improving neurofeedback tasks for rehabilitation (Krakauer and Mazzoni, 2011; Stealey et al., 2024). Both adaptation and *de novo* learning have been observed to contribute to the acquisition of new motor skills and are used as strategies to cope with changing environments or conditions (Costa et al., 2017; Gallego et al., 2017). Although these two modalities have characteristic timescales over which they vary (Krakauer and Mazzoni, 2011; Gallego et al., 2017), it is hard to determine the relative contribution of each when executing motor tasks. For this purpose, brain-computer interfaces (BCIs) have been successfully employed to understand the evolution of neural dynamics when subjects are presented with a diverse range of visuomotor tasks. These tasks often involve introducing perturbations that enable researchers to directly measure which changes in neural dynamics are concomitant with the recovery of task proficiency (Jarosiewicz et al., 2008; Ganguly and Carmena, 2009; Chase et al., 2012; Costa et al., 2017; Golub et al., 2018; Zippi et al., 2022).

BCIs are particularly well suited to understanding the contributions of adaptation and learning in acquiring and modifying motor tasks. In particular, studying neural recordings from the lens of dynamics and neural manifolds has indicated that adaptation often occurs within stable manifolds, whereas learning can result in new dynamics that diverge from the original low-dimensional manifold (Ganguly and Carmena, 2009; Shenoy et al., 2013; Sadtler et al., 2014; Gallego et al., 2017; Vyas et al., 2018; Oby et al., 2019; Yang et al., 2021; Deng et al., 2022; Mitchell-Heggs et al., 2023). For instance, it has been shown that in BCI center-out reaching tasks low-dimensional representations of neural activity are isomorphic with the task itself. Namely, activity corresponding to reaches to radially distributed targets is clustered in low-dimensional space in a circular configuration (Santhanam et al., 2009; Vyas et al., 2018).

Along with insights from BCI studies, reinforcement learning (RL) agents have been proposed as analogs to biological agents (Doll et al., 2012; Lubianiker et al., 2022) as they can be trained to perform similar tasks. Reinforcement learning has been used with considerable success in elucidating the role of reward prediction error in binary decision-making. Indeed this approach has contributed to the development of the reward prediction error theory of dopamine (Montague et al., 1996; Doll et al., 2012). However, the use of RL agents to determine correlates of animal behavior for continuous tasks has remained much more limited. RL agents have yet to be explored as analogs of NHPs performing continuous feedback tasks with perturbations.

The artificial neural networks encoding the policies of RL agents may yield insights into how modifications in activity and connectivity can account for task acquisition and adaptation. Even though RL agents can produce qualitatively similar behavior to animal subjects, it can do so via substantially different architectures and with simplified neural units. Studying which features of the natural and artificial neural dynamics are preserved in response to perturbations in both animal subjects and RL agents can help to establish the validity of the analogy between the two. Moreover, this helps to highlight the different mechanisms operating in both in response to a changing environment.

Here we explore how deformations within a low-dimensional manifold of neural activity can directly account for strategies that compensate for imposed perturbations in a BCI center-out reaching task with rotational perturbations. We compare results from two NHP subjects and a RL agent trained in a virtual center-out reaching task. Our results indicate that there is a distinctive signature for adaptation in the NHP subjects, as the low-dimensional manifold is preserved and the deformations within this manifold directly compensate for the imposed rotational perturbations.

In this paper we demonstrate that rapid NHP adaptation is achieved via exact compensation by geometric rotation of the underlying neural activity. We show that ANN-based RL agents can leverage the same low-dimensional isomorphic structure as NHPs when performing the same task. In comparing how, we establish that, in spite of the shared isomorphism in NHPs and RL agents, maximizing reward with very similar trajectories after perturbations can proceed by very different mechanisms. This in turn is suggestive of the very limited role that plasticity needs to play for short-term adaptation. Namely, the RL agent changes connection strengths substantially to maximize rewards, which leads to modifying the underlying manifold. In contrast, the preserved manifold of the NHPs suggests that neural connection strengths largely remain unchanged after adapting to the perturbation. We describe experimental and computational methods in the following section. Section 3 describes and compares the results from experiments and simulations, and Section 4 discusses the results with emphasis on the interplay between adaptation and learning. Finally, we offer concluding remarks as well as potential future research directions.

## 2 Methods

### 2.1 Non-human primate neurofeedback task

Two male Rhesus macaque (Macaca mulatta) monkeys were trained in a BCI center-out reaching task. The cursor was controlled by volitional modulation of action potential (“spiking”) activity from a population of recorded neurons. We recorded spiking activity using a chronic microelectrode array (MEA) comprising of tungsten wires (diameter 35μ*m*) (Innovative Neurophysiology, Inc., Durham NC) into the primary motor (M1) and pre-motor (PMd) cortical areas of the left hemisphere. Subject A was implanted with 64 electrodes and Subject B with 128 electrodes. The number of independent recorded units varied in the ranges 22–50 and 51–136 for Subjects A and B, respectively. A more detailed description of surgical and training procedures can be found in previous work by Stealey et al. (2024).

The center-out reaching task consisted of driving the cursor from a central location to one of eight different targets radially distributed with a uniform angular separation of 45 degrees and fixed distance from the center target. Successful “hold” periods after movement of the cursor to the cued peripheral target, referred to as a “reach,” was reinforced with a fluid reward. The recorded neural activity was mapped to a control signal that updated cursor velocity in each time bin (100 ms) using a Kalman filter paradigm that can be expressed mathematically as (Equation 1):


(1) $$x_{t+1}=Ax_{t}+Ky_{t}$$

Where the vector **x** comprises the cursor position, velocity and a constant term, the vector **y** contains the temporally averaged spiking activity over 100 ms windows, the matrix **A** represents a dynamics matrix that remains constant across experimental sessions, and the matrix **K**, called the Kalman gain, directly maps neural activity to cursor dynamics and is estimated at the beginning of every experimental session. The Kalman filter is fit using neural activity recorded during passive observation of the cursor moving along straight trajectories to each of the peripheral targets. This neural activit is obtained at the beginning of each BCI session. This procedure has been extensively described in previous work (Gowda et al., 2014; Stealey et al., 2024 and references therein). Mathematically, the Kalman filter approach formulates spiking activity as linearly dependent on the state of the cursor (Equations 2, 3):


(2) $$x_{t+1}=\tilde{A}x_{t}+w_{t}\;;\;w_{t}\sim N(0,W)$$

(3) $$y_{t}=Cx_{t}+q_{t}\;;\;q_{t}\sim N(0,Q)$$

**Figure 1:**
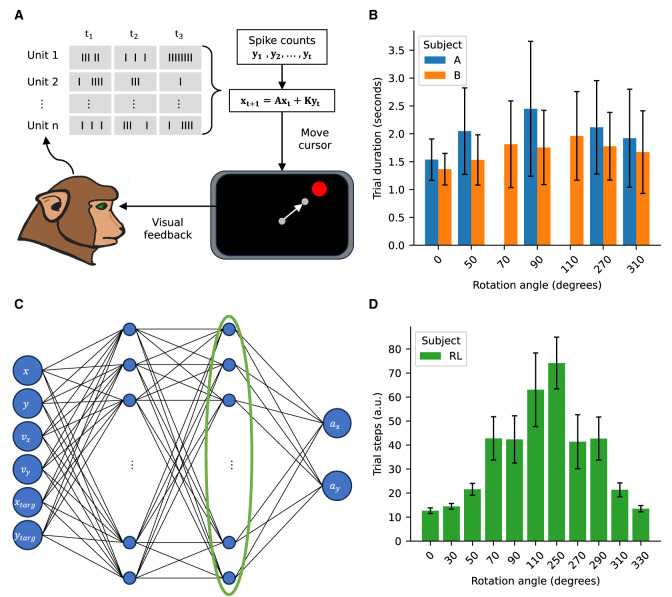
Experimental approach schematic. **(A)** Schematic of the BCI center-out reaching task. Spiking activity recorded from M1 and PMd areas is used to control the cursor. The recorded spikes are decoded via a Kalman filter to control the cursor velocity. **(B)** Trial durations for the NHPs for the imposed rotations. **(C)** Schematic of the policy network for the RL agent. The value network has the same architecture. The activity of the last hidden layer, highlighted by the green ellipse, is used for the manifold analysis as an analog to the firing rates driving the cursor in the NHP task. **(D)** Trial steps for the RL agent for the imposed rotations. Error bars represent standard deviation with N_*A*_ = (21,080; 10,559; 9,593; 8,968; 12,032), N_*B*_ = (25,050; 9,420; 6,710; 10,100; 4,255; 10,716; 9,886), N_*RL*_ = (100; 1,000; 1,000; 1,000; 720; 154; 88; 680; 931; 1,000; 1,000), where the subscripts indicate the subject.

The matrix $\tilde{\text{A}}$ gives prescribed cursor kinematics and the matrix **C** is fit from data collected at the beginning of each session (when the cursor is moving along prescribed trajectories). The matrices **W** and **Q** define the covariances of Gaussian noise processes. After obtaining said matrices, the cursor position can be estimated from neural observations and compared to its actual (prescribed) position. The Kalman gain, **K**, then determines the weight given to a model relative to the weight given to observations in updating predictions of the state **x**. The gain is computed from prediction error covariance, the measurement matrix, **C**, and the measurement noise covariance (Simon, 2006). The new dynamics matrix can then be readily computed as $A=(I-KC)\tilde{A}$. A schematic view of the cursor control is depicted in Figure 1A.

After the Kalman gain is estimated the subjects complete a baseline block during which they proficiently reach all the targets. Subsequently, a visuomotor rotation (VMR) perturbation is introduced by multiplying the Kalman gain by a block diagonal matrix (Equation 4). Said matrix has two blocks describing an imposed rotation of angle θ (Equation 5) corresponding to rotations of position and velocity components of the vector **x**.


(4) $$x_{t+1}=Ax_{t}+R(\theta )Ky_{t}$$

(5) $$R(\theta )=\left[\begin{array}{cc} \cos(\theta ) & -\sin(\theta )\\ \sin(\theta ) & \cos(\theta ) \end{array}\right]$$

We imposed both clockwise and counter-clockwise decoder rotations with magnitudes ranging between 50° and 110°. After the rotation was imposed in the decoder both subjects compensated and were able to complete the task (Stealey et al., 2024). Time to complete successful reaches were comparable across the different conditions (Figure 1B).

### 2.2 Reinforcement learning agent virtual center-out reaching task

To better understand the underpinnings of the adaptation achieved by the NHP subjects we created a RL analog to the center-out reaching task. We used a Proximal Policy Optimization (PPO) algorithm as implemented by the stable baselines 3 library (https://stable-baselines3.readthedocs.io/en/master/modules/ppo.html) (Schulman et al., 2017). The policy network contained two fully connected layers with 128 units each and was trained to optimize for output velocities to drive the cursor toward the targets (Figure 1C). The reward function penalized increases in the distance to the target and rewarded decreasing the distance to the target. Additionally the square of the magnitude of the policy velocities was penalized so as to enforce smooth motion. Finally a large reward was granted upon reaching a target. Mathematically, the update (Equations 6, 7) and reward (Equation 8) can be expressed as follows:


(6) $$\begin{array}{l} {\text{v}}_{t+1}^{cursor}=0.1\cdot {\text{v}}_{t}^{cursor}+\text{action} \end{array}$$

(7) $$\begin{array}{l} {\text{x}}_{t+1}^{cursor}={\text{x}}_{t}^{cursor}+\Delta t\cdot {\text{v}}_{t}^{cursor} \end{array}$$

(8) $$\text{reward}=\{\begin{array}{ll} 20-0.5\cdot \Delta d_{ct}-|action{|}^{2} & \text{if reached target}\\ -0.5\cdot \Delta d_{ct}-|action{|}^{2} & \text{otherwise} \end{array}$$

Where, **x**^**cursor**^ and **v**^**cursor**^ are the cursor's position and velocity, respectively; and Δ*d*_*ct*_ is the change in the distance between the cursor and the target given the **action** generated by the RL agent, with euclidean norm |**action**|. In our implementation Δ*t* = 1. This function rewards getting closer to the target for each action and provides a substantial reward once the target is reached, while also penalizing large changes in velocity. This serves to promote smoother trajectories and avoid the agent just shooting to the target in one step.

We subsequently emulated the effect of the visuomotor rotation for the NHP subjects by creating an alternative rotated environment whereby the velocities computed by the agent where multiplied by a rotation matrix like the one in Equation 5. The agent's artificial neural network was retrained to complete the task in the new rotated environment for a sufficient number of epochs as to achieve reliable success for all targets (Figure 1D).

### 2.3 Low-dimensional manifold extraction via principal component analysis (PCA)

We extracted the low-dimensional activity for both the NHPs and the RL agent via principal component analysis (PCA). PCA is done by performing the singular value decomposition on the data matrix where each column is an observation either of firing rate (activation) at a time point after substracting the mean observation for the NHP (RL) neural activity. PCA can provide a linear manifold spanned by a subset of the principal component vectors. In this study we focus on the two-dimensional linear subspace since the task under consideration is two-dimensional and in this subspace activity is isomorphic with task.

## 3 Results

### 3.1 Low-dimensional representations of neural activity are isomorphic with the center-out reaching task

**Figure 2:**
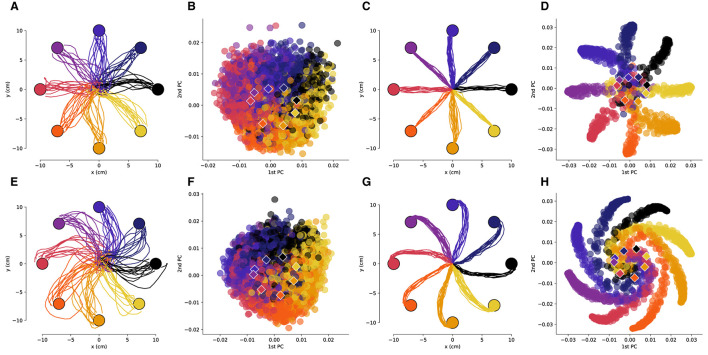
The low-dimensional neural activity for Subject B is isomorphic with the task geometry. **(A)** Representative trajectories from one session for Subject B before rotation, and **(B)** the corresponding PCA representation of the driving spiking activity. **(C)** Representative trajectories for RL agent before rotation, and **(D)** the corresponding PCA representation of the last fully connected layer of the ANN. **(E–H)** Results after a –50 degree rotation is imposed, plots follow the same sequence as in **(A–D)**. The circles represent the target locations, the PCA values are colored by the corresponding target and the centroids of each of the clusters are depicted by the rhombi.

**Figure 3:**
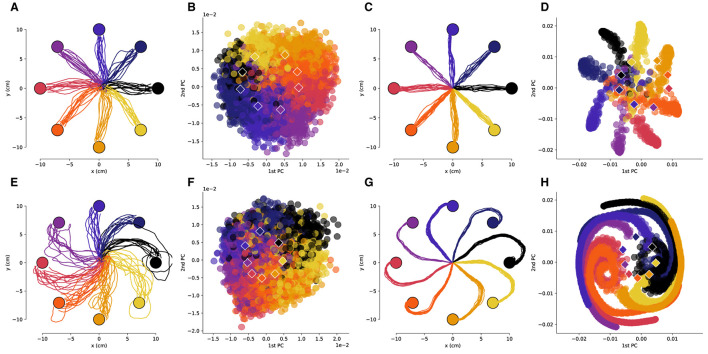
The low-dimensional neural activity for Subject B is isomorphic with the task geometry after large rotations. **(A)** Representative trajectories from one session for Subject B before rotation, and **(B)** the corresponding PCA representation of the driving spiking activity. **(C)** Representative trajectories for RL agent before rotation, and **(D)** the corresponding PCA representation of the last fully connected layer of the ANN. **(E–H)** Results after a 110 degree rotation is imposed, plots follow the same sequence as in **(A–D)**. The circles represent the target locations, the PCA values are colored by the corresponding target and the centroids of each of the clusters are depicted by the rhombi.

We obtained low-dimensional representations of the neural activity during the reaching task using principal component analysis (PCA). As was observed in previous works (Santhanam et al., 2009), the neural activity in a low-dimensional space is isomorphic with the center-out reaching task (Figures 2A, B, 3A, B). This isomorphism is maintained even in the presence of the rotation perturbation (Figures 2E, F, 3E, F). Even though the reach trajectories are visibly affected by the imposed rotation (Figures 2A, E, 3A, E), the PCA representation remains isomorphic with the task (Figures 2B, F, 3B, F). The PCA was performed over all data (with and without rotation) so that the PCA basis is the same in all conditions. Interestingly, in the two-dimensional space the centroids of the PCA clusters for each target are rotated in the opposite direction of the imposed rotation.

We then compared the underlying neural manifolds for the NHP subjects and the artificial neural network (ANN) of our RL agent. We performed a similar PCA analysis for the activations of the last fully connected layer of the RL policy network. We obtained trajectories that were qualitatively similar before and after imposing a rotation to those from the NHP subjects (Figures 2C, G, 3C, G). As was the case for the NHP data, the ANN activations have a low-dimensional PCA representation that is isomorphic to the task geometry (Figures 2D, 3D). However, after imposing the rotation, the geometry of the low-dimensional representation of the activations is substantially different from before imposing the rotations, in stark contrast to the results from the NHP data (Figures 2F, H, 3F, H).

**Figure 4:**
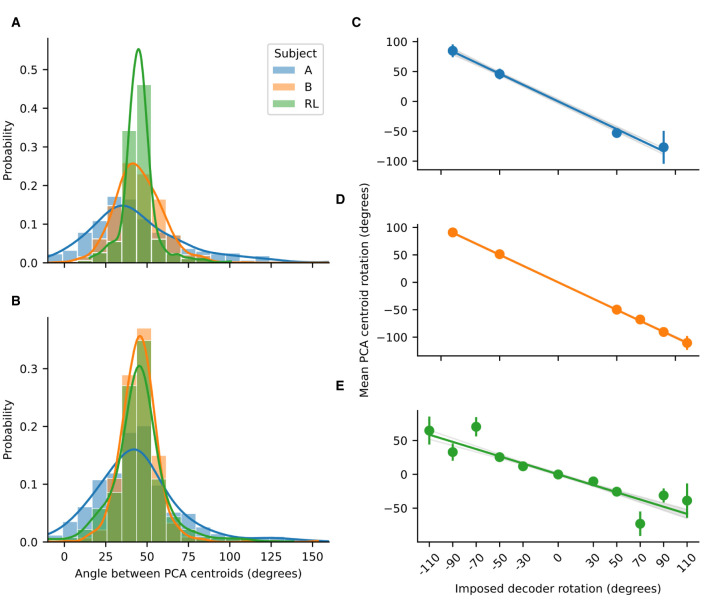
Probability distributions for the angles between adjacent PCA centroids. **(A)** Probability distributions before imposing rotation and **(B)** after imposing rotation, for NHP subjects A and B, and for the RL agent. Lines are the kernel density estimates to smooth the distributions. The distributions where approximately gaussian with a mean of 45 degrees. Rotations in low-dimensional space compensate for imposed decoder rotation. Linear regressions for the mean centroid angular displacement are shown for **(C)** Subject A (*R*^2^ = 0.95, slope = –0.927); **(D)** Subject B (*R*^2^ = 0.996, slope = –1.0038); and **(E)** the RL agent (*R*^2^ = 0.752, slope = –0.5322). For the NHPs, the rotations in low-dimensional space are of almost the same magnitude as the imposed decoder rotation. Error bars represent standard deviation. For all regressions *p* < 0.001.

For both the NHP data and the RL simulations, the angles between adjacent PCA centroids approximate the angles between the targets (Figure 4). Substantial differences in the standard deviations were observed, with the tightest distribution being the one for the RL agent and the broadest for Subject A (who had fewer spiking units in the decoder). Thus, we see that projection of neural activity into low-dimensional PC space not only preserves the geometry of the task but also approximately preserves the angular distances for the different reaching directions.

### 3.2 Rotations in a low-dimensional space compensate for imposed decoder rotations

As can be noted qualitatively in Figures 2B, D, the imposed decoder rotations shift the PCA clusters corresponding to each target. We quantified the angular displacement of each cluster over all experimental sessions revealing that it is of opposite sign and equal magnitude to the imposed decoder rotation (Figure 4). Linear regressions indicate that for the NHPs the rotation in the low-dimensional neural space almost exactly cancels out the imposed decoder rotation. In contrast, for the RL agent the rotation in low-dimensional space exhibits some non-linear behavior as a function of imposed decoder rotation and the resulting slope deviates farther from negative unity. Nevertheless, in all cases the linear trends robustly indicate that neural activity not only has a low-dimensional geometry that is similar to the task, but is also transformed in a manner that directly compensates for the geometry of imposed perturbations.

### 3.3 Low-dimensional manifold is preserved after rotation for NHPs

**Figure 5:**
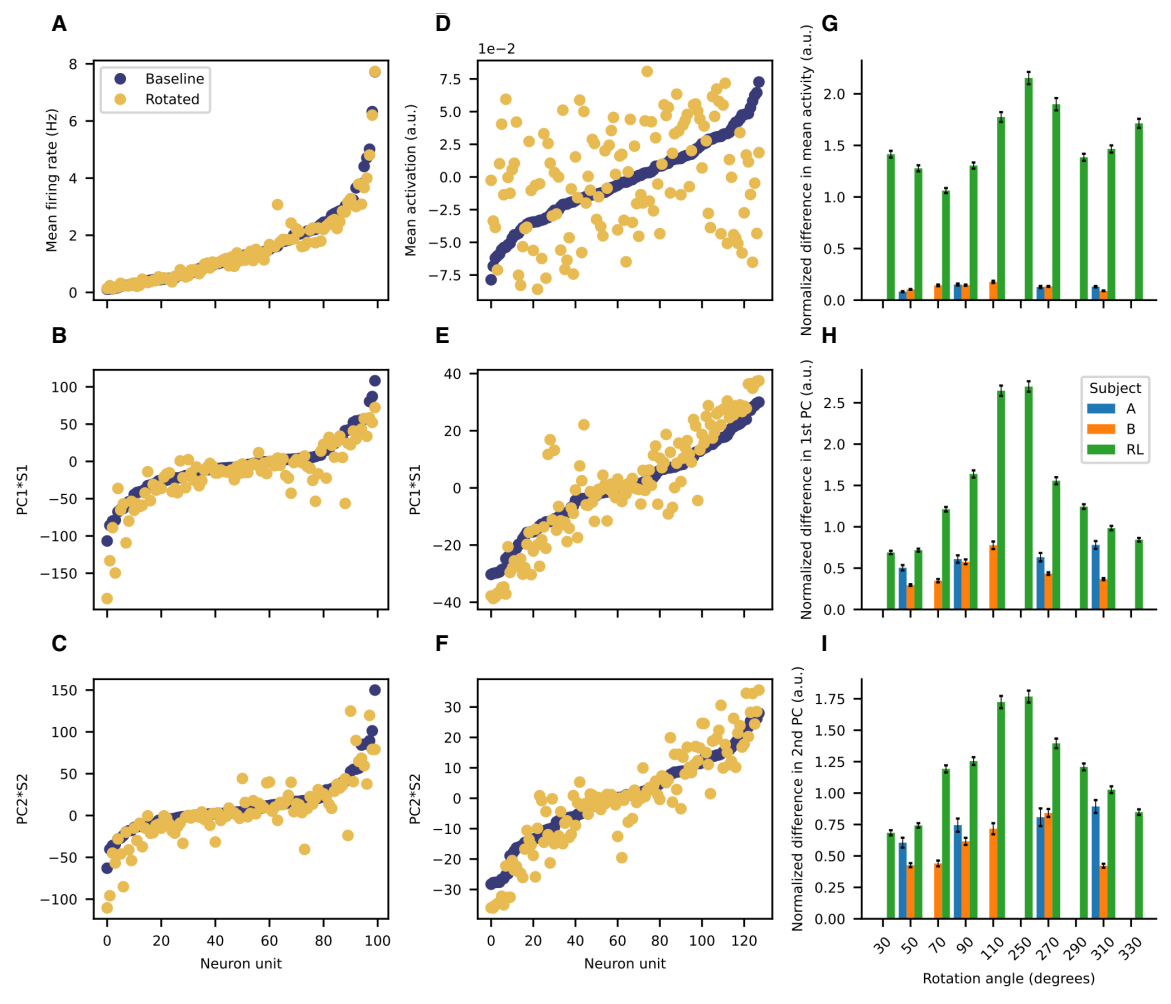
Low-dimensional manifold is preserved after rotation for the NHPs but not for the RL agent. Representative examples of the mean firing rate **(A)** the first PC **(B)** and the second PC **(C)** scaled by their respective singular values before and after rotation for Subject B. Representative examples of the mean activation **(D)** the first PC **(E)** and the second PC **(F)** scaled by their respective singular values before and after rotation for the RL agent. Bar plot summaries of the normalized differences in: **(G)** mean activity **(H)** scaled first principal component **(I)** scaled second principal component. Error bars represent the standard error of the mean. For the imposed rotations for which all subjects have data (50, 90, 270, and 310 degrees), the means are significantly different (*p* < 0.001, one-way ANOVA).

We then investigated whether the low-dimensional PCA manifold was preserved after rotating the decoder. The results for the previous subsection considered the same PCA basis for all the data (with and without rotations), but such a strategy is sub-optimal if the underlying manifolds before and after imposing the perturbations are distinct. Thus, we consider PCA performed separately for data before and after rotations. The recorded neural activity for the NHPs is remarkably stationary throughout the session (Figure 5). The mean firing rates are quite similar before and after the perturbation (Figure 5A). Moreover, the first and second principal component vectors are also quite similar before and after the rotation is imposed (Figures 5D, G). In contrast, activations for the ANN of the RL agent have significantly different means before and after rotation (Figure 5B), although the principal component vectors do not deviate so markedly (Figures 5E, H). To compare accross sessions, we normalize differences in firing rates as follows (Equation 9):


(9) $$\begin{array}{l} d_{i}=\frac{{\bar{f}}_{b_{i}}-{\bar{f}}_{r_{i}}}{\frac{1}{N}\overset{N}{\underset{i=1}{\sum }}\left({\bar{f}}_{b_{i}}\right)} \end{array}$$

Where *d*_*i*_ is the normalized difference for unit *i* and ${\bar{f}}_{b_{i}}$ is the temporally averaged baseline firing rate and ${\bar{f}}_{r_{i}}$ is the temporally averaged firing rate after rotation, and the N is the number of units for a given session (so that the differences are normalized by the session mean baseline activity).

**Figure 6:**
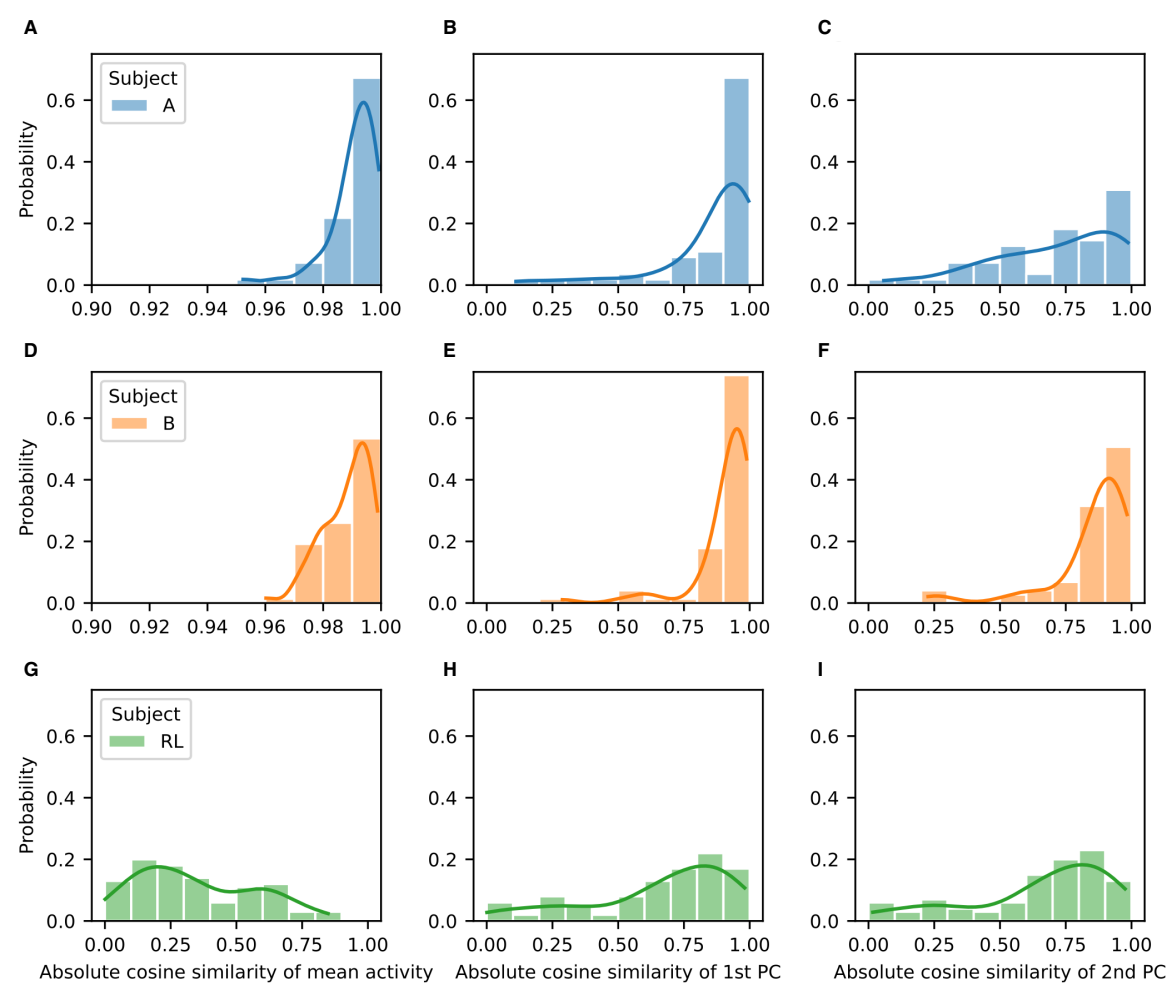
Low-dimensional manifold for baseline and perturbation activity is highly similar for the NHPs but not for the RL agent. Probability distributions of absolute cosine similarity for the mean activity **(A, D, G)**, first principal component **(B, E, H)**, and second principal component **(C, F, I)** before and after rotation. Results correspond to Subject A **(A–C)**, Subject B **(D–F)**, and the RL agent **(G–I)**.

We quantified the difference both in the mean firing rates (activations) and in the two first principal components by using the absolute value of the cosine similarity for the corresponding vectors before and after rotation (Figure 6). As shown in the representative example, for the NHPs the mean firing rate and the two first principal components are quite similar. They show distributions that are highly skewed toward values close to unity (Figure 6, left and center columns). In contrast, the RL agent displays significant differences in the mean activation value, with no values near unity (Figure 6, right column). Distributions for the difference of the principal components before and after rotation are skewed in a similar fashion as those of the NHP, but with peaks closer to a cosine similarity of 0.9 rather than unity. These results suggest that the low-dimensional manifold is much better preserved in the case of the NHPs than in the case of the reinforcement learner.

## 4 Discussion

The results are consistent with the intuitive notion that the NHPs compensate for decoder rotation by re-aiming their reaches at an angle that counteracts the decoder perturbation angle. Such a strategy could be achieved by simply generating similar neural activity as before the perturbation is introduced, but in a different context. Namely, if a 45 degree rotation is imposed, the NHPs could generate the same activity that helped them reach a target located at –45 degrees under the original decoder. Such a strategy would preserve the underlying low-dimensional manifold. We call this approach *adaptation*, as it leverages existing neural pathways displaying dynamics in a stable manifold. NHPs seem to adapt their existing neural dynamic repertoire to the changing decoder.

In contrast, reinforcement learning algorithms rely on updating the weights between the ANN's layers. Even though we recreated qualitatively accurate trajectories in our virtual environment, the trajectories generated in response to imposed rotations were generated by a substantially different mechanism. This mechanism changes the mean activity of the artificial neurons and modifies the underlying manifold, rather than re-purposing the existing dynamics. We speculate that this substantial modification of the neural manifold is the hallmark of extensive changes in connectivity. These changes can be understood as *learning* in the sense that novel strategies and dynamics emerge.

In the absence of direct observations of the connectivity in behaving animals, it is hard to demonstrate that adaptation (rather than learning through synaptic changes) is the dominant mechanism allowing NHPs to quickly, flexibly and reversibly respond to perturbations. However, preservation of both the neural manifold and the mean firing for each unit is suggestive of higher level planning that directs commands through reliable and well-established pathways. From a biological perspective, it stands to reason that adaptation of motor tasks should not demand extensive changes in connectivity arising from the demands of a dynamic environment.

## 5 Conclusion, limitations, and future scope

We presented evidence that the low-dimensional manifold of the neural dynamics of NHPs during a center-out reaching task preserves the geometry of the task and exhibits deformations that almost exactly counteract imposed decoder rotations. The preservation of the low-dimensional manifold is consistent with the adaptation of a well-established motor repertoire to novel challenges. In contrast, a reinforcement learner agent that originally has dynamics that are also isomorphic to the task substantially modifies its manifold in response to imposed rotations to maximize its reward.

For the present study, we utilized a traditional Kalman filter approach to decode a cursor control signal from neural firing rates and drive a cursor for real time feedback. This approach has the advantage of being parsimonious, thus having low training data requirements. However, recent developments in neural decoders using deep and convolutional neural networks (Glaser et al., 2020; Filippini et al., 2022; Borra et al., 2023) can result in improved performance. Moreover, non-linear decoders may allow for better reconstruction of the natural task-related manifold. Future work could utilize these improved decoding approaches to elucidate whether they not only improve baseline decoding but also allow for better and more rapid adaptation.

A more complete exploration of the adaptation strategy in NHPs would require recording from other brain regions, including regions that are not directly used by the decoder. This would allow us to observe where the isomorphism breaks down and what activity can be directly correlated to the adapting strategies. In addition, future work should focus on refining the RL approach by exploring model-based RL algorithms that may enable higher order planning and/or imposing constraints such as preserving the activity manifold in some of the ANN layers.

## Data availability statement

The raw data supporting the conclusions of this article will be made available by the authors, without undue reservation.

## Ethics statement

The animal study was approved by the University of Texas at Austin Institutional Animal Care and Use Committee. The study was conducted in accordance with the local legislation and institutional requirements.

## Author contributions

RO-O: Conceptualization, Formal analysis, Investigation, Methodology, Validation, Visualization, Writing – original draft, Writing – review & editing. YZ: Data curation, Formal analysis, Investigation, Methodology, Writing – original draft, Writing – review & editing. HS: Data curation, Methodology, Writing – original draft, Writing – review & editing. H-YL: Data curation, Methodology, Writing – original draft, Writing – review & editing. EC-H: Data curation, Methodology, Writing – original draft, Writing – review & editing. SS: Conceptualization, Funding acquisition, Methodology, Project administration, Resources, Supervision, Writing – original draft, Writing – review & editing.
